# Changes of Circulating Extracellular Vesicles from the Liver after Roux-en-Y Bariatric Surgery

**DOI:** 10.3390/ijms20092153

**Published:** 2019-04-30

**Authors:** Gersina Rega-Kaun, Dorothea Ritzel, Christoph Kaun, Benjamin Ebenbauer, Barbara Thaler, Manfred Prager, Svitlana Demyanets, Johann Wojta, Philipp J. Hohensinner

**Affiliations:** 1 Department of Internal Medicine II, Medical University of Vienna, A-1090 Vienna, Austria; gersina.rega-kaun@meduniwien.ac.at (G.R.-K.); n1242214@students.meduniwien.ac.at (D.R.); christoph.kaun@meduniwien.ac.at (C.K.); benjamin.ebenbauer@hotmail.com (B.E.); barbara.thaler@gmx.net (B.T.); svitlana.demyanets@meduniwien.ac.at (S.D.); philipp.hohensinner@meduniwien.ac.at (P.J.H.); 2 5th Medical Department for Endocrinology and Rheumatology, Wilhelminenhospital, A-1160 Vienna, Austria; 3 Ludwig Boltzmann Cluster for Cardiovascular Research, A-1090 Vienna, Austria; 4 Department of Surgery, Hospital Oberwart, A-7400 Oberwart, Austria; manfred.prager@gmx.at; 5 Department of Surgery, Hospital Hietzing, A-1130 Vienna, Austria; 6 Department of Laboratory Medicine, Medical University of Vienna, A-1090 Vienna, Austria; 7 Core Facilities, Medical University of Vienna, A-1090 Vienna, Austria

**Keywords:** extracellular vesicle, bariatric surgery, obesity

## Abstract

Circulating extracellular vesicles are small particles enclosed by a phospholipid bilayer. Vesicles deriving directly from the cellular membrane by an active budding process retain cell origin specific proteins and RNA. These vesicles carry pathophysiological information from their parental cell and hold the potential to allow analysis of organs without the need for a biopsy. We included in our study 27 patients undergoing bariatric surgery. Hepatic extracellular vesicles were determined by flow cytometry. mRNA specific for hepatic cellular origin was determined in the extracellular vesicle fraction using qPCR. Surgery led to a massive reduction of weight and overall hepatic stress as determined by alanine transaminase (ALT), aspartate transaminase (AST) and γ-glutamyltransferase (GGT). Total extracellular vesicle numbers were reduced after bariatric surgery. Liver specific vesicles identified by HepPar1 or asialoglycoprotein receptor (ASGPR) were significantly reduced after bariatric surgery in both AnnexinV^+^ and AnnexinV^−^ subgroups. When analyzing circulating liver-specific mRNAs, we found reduced levels of these mRNAs after surgery even though total circulating RNA remained unchanged. We conclude that circulating hepatic extracellular vesicles are detectable in samples from patients undergoing gastric bypass surgery. These vesicles are reduced after a reduction of hepatic stress also observed with classic liver enzyme measurements. We conclude that ASGPR or HepPar positive vesicles hold the potential to serve as liver specific vesicle markers.

## 1. Introduction

Extracellular vesicles are small particles heterogeneous in size ranging from 20 nm to 2000 nm [1]. Originating directly from cells they are enclosed by a phospholipid bilayer and released into extracellular medium both in vivo and in vitro. The smallest vesicles are exosomes with a usual size below 150 nm. Exosomes are formed by fusion of an organelle of the endocytic pathway with the plasma membrane [2]. Extracellular vesicles between 200 nm and 900 nm derive directly from the cellular membrane of a cell by an active budding process [3]. This budding process can be related to apoptosis, however it is not a prerequisite for extracellular vesicle formation [4]. As these extracellular vesicles derive from the cellular membrane, “parent” cells of vesicles can be easily identified by staining for specific proteins. In addition, vesicles can carry information in the form of proteins or RNA including mRNA or miRNA from the parental cell and serve as non-contact dependent signal transduction mediators [5]. Finally, apoptotic bodies form the largest group in size of the extracellular vesicles ranging from 1000–2000 nm. This population is rapidly engulfed by phagocytic cells [6]. Recently, the International Society for Extracellular Vesicles (ISEV) suggested a nomenclature for extracellular vesicles [7]. In this position paper the ISEV endorses the term “extracellular vesicle” as the generic term for particles naturally released from the cell. Assigning an EV to a particular biogenesis pathway remains extraordinarily difficult and operational terms for EV subtypes should therefore be considered.

Given tissue specific identification, extracellular vesicles might be suitable to gather information about organs otherwise only accessible by biopsies [8]. In addition, extracellular vesicles might be increasingly released under stress situations. Hepatic microvesicles were already previously described to be associated with increased stress. Povero et al. recently reported increased asialoglycoprotein receptor (ASGPR)^+^ circulating microvesicles in a rat model of liver injury and fatty liver disease [9,10]. In addition, hepatic microvesicles identified as being carbamoyl phosphate synthetase 1 (CPS1) positive can be detected in liver injured animals [11].

Obesity is considered to be a global epidemic associated with numerous comorbidities including cardiovascular disease and liver dysfunction [12]. Current guidelines recommend gastric bypass surgery at a body mass index (BMI) >40 kg/m^2^ or a BMI >35 kg/m^2^ with secondary disease [13]. This surgical intervention results in massive weight loss within the first year after surgery [14]. The weight loss is associated with dramatic changes such as a reduced inflammatory state and reduced hepatic stress [15,16,17].

The aim of our study was to determine if hepatic extracellular vesicles and their content can be detected in human patient samples. Furthermore, we wanted to evaluate if circulating extracellular vesicles change upon physiological changes using a cohort of morbidly obese patients undergoing gastric bypass surgery and subsequent dramatic weight loss.

## 2. Results

Bariatric surgery led to a drastic reduction of weight (122.64 kg before 78.11 kg after surgery; *p* < 0.0001), and BMI (43.25 before 27.5 after surgery; *p* < 0.0001). Of the three classically evaluated liver function markers aspartate transaminase (AST), alanine transaminase (ALT) and γ-glutamyltransferase (GGT), we found that both ALT and GGT were significantly reduced. AST showed a non-significant reduction one year after surgery. Mean values for high density lipoprotein (HDL) and low density lipoprotein (LDL) before the intervention were close to the values recommended by the National Lipid Association of >40 mg/dl (men) and >50 mg/dl (female) for HDL and <100 mg/dl for LDL respectively (Table 1) [18]. Of note, the seven patients taking statins had an average of 95.7 ± 40.2 mg/dL of LDL. In addition, C-reactive protein (CRP) values were reduced after bariatric surgery. Values for the classical adipokine plasminogen activator inhibitor 1 (PAI-1) were similarly reduced after bariatric surgery. Overall, all depicted parameters improved after bariatric surgery in spite of a reduced medication scheme (Table 1). Using this patients’ cohort, the aim of our study was to determine the possibility of detecting hepatic extracellular vesicles in the circulation and to investigate if those vesicles would be influenced by Roux-en-Y gastric bypass (RYGBP) surgery. Vesicles were measured using flow cytometry. We used beads for size determination of vesicles and determined the amount of extracellular vesicles in the size range of 200–900 nm terming them in accordance with the guidelines [7] as medium EVs. 

**Figure 1:**
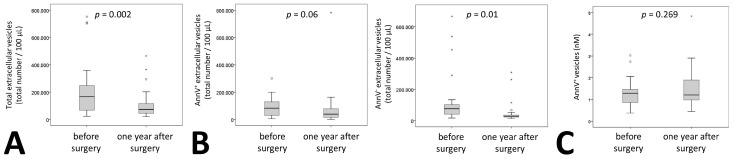
Extracellular vesicle dynamics in patients undergoing bariatric surgery. (**A**) Total medium extracellular vesicle content was determined by flow cytometry analysis in citrated plasma of 27 patients before and one year after surgery. Results are given as total number of extracellular vesicles per 100 µL plasma. (**B**) Phosphatidylserine positive medium extracellular vesicles in citrated plasma of 27 patients before and one year after surgery were identified by staining with AnnexinV (AnnV), the remaining medium extracellular vesicles were defined as AnnV^–^. Numbers of positive and negative medium extracellular vesicles were evaluated using flow cytometry. Results are given as total number of extracellular vesicles per 100 µL plasma. (**C**) Phosphatidylserine positive medium extracellular vesicles were analyzed in 26 patients using an ELISA as indicated under Materials and Methods. Values are given as AnnexinV^+^ extracellular vesicles in nM. For (A–C) a Wilcoxon test was used. *p* ≤ 0.05 was considered significant.

Similar to these classical laboratory parameters, medium EVs were significantly reduced one year after surgery by 59% (Figure 1A). When analyzing phosphatidylserine (PS)^+^ and PS^–^ vesicles by AnnexinV staining we found that the reduction was significant only for AnnexinV^–^ vesicles. Levels of Annexin V^+^ vesicles were reduced by 56% with a *p* = 0.06 whereas AnnexinV^–^ vesicles showed a statistically significant reduction of 62% one year after surgery (Figure 1B). No reduction for AnnexinV^+^ vesicles was observed using a specific ELISA for Annexin V^+^ extracellular vesicles (Figure 1C).

**Figure 2:**
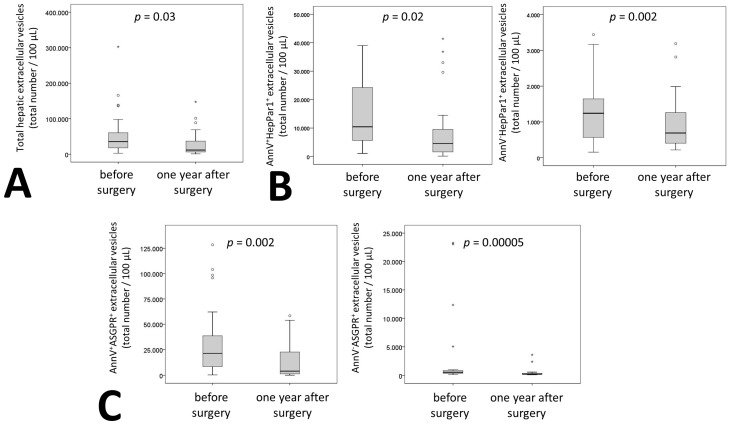
Extracellular hepatic vesicle dynamics in patients undergoing bariatric surgery. (**A**): Total hepatic medium extracellular vesicles in citrated plasma of 27 patients before and one year after surgery were defined as HepPar1^+^, asialoglycoprotein receptor (ASGPR)^+^, and HepPar1^+^ASGPR^+^ medium extracellular vesicles using flow cytometry. HepPar1^+^ (**B**) or ASGPR^+^ (**C**) medium extracellular vesicles in citrated plasma of 27 patients before and one year after surgery were evaluated for their AnnV staining pattern. Absolute numbers of extracellular vesicles positive for AnnV^+^ and AnnV^–^ were determined by flow cytometry. Values are given per 100 µL of plasma. For (A–C) a Wilcoxon test was used. *p* ≤ 0.05 was considered significant.

Hepatocyte specific medium EVs were identified using HepPar1 and ASGPR. Furthermore, these hepatic extracellular vesicles were discriminated by the presence or absence of phosphatidylserine (PS). We found that both ASGPR^+^AnnV^–^ and HepPar^+^AnnV^–^ vesicle subsets correlated at baseline with PAI-1 levels (r = 0.398, *p* = 0.04 for ASGPR^+^AnnV^–^ and r = 0.395, *p* = 0.04 for HepPar^+^AnnV^–^). Interestingly, this was not the case for PS^+^ medium EVs. Furthermore, at baseline, diabetic patients had significantly higher total medium EVs (*p* = 0.026). However, no significant changes in hepatic vesicles were observed. All hepatocyte extracellular vesicles together showed a significant reduction after bariatric surgery of 68%. This was observed for single positive and double positive medium EVs (Figure 2A). In contrast to the overall medium EV population, we observed reduced levels for both Annexin V^+^ and Annexin V^–^ hepatic medium EVs of 50% for HepPar^+^AnnV^+^, 29% for HepPar^+^AnnV^–^, 81% for ASGPR^+^AnnV^+^, and of 61% for ASGPR^+^AnnV^–^ (Figure 2B,C). Of note, reduction of extracellular vesicle number did not correlate with changes in clinical parameters including AST, ALT and GGT besides Annexin V^+^ ASGPR^+^ extracellular vesicles. Interestingly, Annexin V^+^ ASGPR^+^ extracellular vesicle relative reduction one year after surgery correlated with a relative reduction of ALT (r = 0.403, *p* = 0.037). Individual patient changes in medium EV fractions are displayed in Supplementary Figure S1. To further characterize ASGPR^+^ extracellular vesicles we determined the amount of CD63^+^ASGPR^+^ vesicles that could derive from an exosomal pathway in seven patients. Of the ASGPR^+^ medium extracellular vesicles, 18.4% ± 8% stained positive for CD63 suggesting that the majority of medium EVs does not originate from an exosomal pathway. 

**Figure 3:**
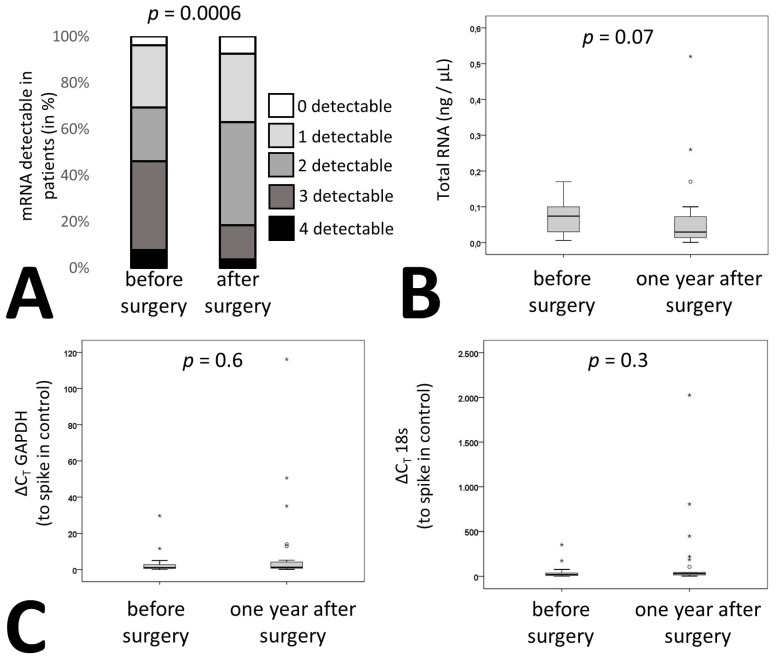
Liver specific RNA patterns in patients undergoing bariatric surgery. (**A**) mRNA was isolated from circulating EVs from citrated plasma of 27 patients before and one year after surgery as indicated in the supplementary methods. qPCR was performed for acyl-coenzyme-A-oxidase-1 (ACOX), acyl-coenzyme-A-dehydrogenase (ACADM), hydroxyacyl-coenzyme-A-dehydrogenase (HADH) and 3-hydroxybutyrate-dehydrogenase (BDH). Patients were stratified depending on the number of detectable mRNAs. (**B**) RNA was isolated from circulating EVs from 27 bariatric surgery patients before and one year after surgery as indicated in Methods and the quantity was determined. (**C**) Relative expression levels of glyceraldehyde-3-phosphate-dehydrogenase (GAPDH) and 18s from circulating EVs were determined in 27 bariatric surgery patients before and one year after surgery as indicated in Materials and Methods. Values given are relative to a spike in control. For (A) statistical significance was calculated using a chi-square test. (B, C) statistical significance was calculated using Wilcoxon test. *p* ≤ 0.05 was considered significant.

In addition to membrane proteins, the cargo of these vesicles can also be used to identify the origin of extracellular vesicles. We therefore measured the signature of liver specific mRNAs in RNA isolated from the pellet of the extracellular vesicle fraction of patients’ plasma after centrifugation as indicated in Materials and Methods. Circulating extracellular vesicle mRNA signature of four hepatic proteins, namely straight-chain-acyl-coenzyme-A-oxidase-1 (ACOX), acyl-coenzyme-A-dehydrogenase (ACADM), long-chain-L3-hydroxyacylcoenzyme-A-dehydrogenase (HADH) and 3-hydroxybutyrate-dehydrogenase (BDH) were influenced by bariatric surgery. Whereas 46% of patients had all four or three of four mRNAs detectable in circulatory extracellular vesicles before surgery only 18% of patients were tested positive for four or three mRNAs after surgery (Figure 3A, *p* = 0.0006). Overall, there was no change in extracellular vesicle-circulating mRNA amounts before and one year after bariatric surgery as confirmed by measurement of total RNA in all patient samples (Figure 3B). In addition, two housekeeping genes, namely 18s RNA and glyceraldehyde-3-phosphate-dehydrogenase (GAPDH) were readily detectable in the extracellular vesicle fraction of all samples and remained at similar levels when adjusting to an input control (Figure 3C).

## 3. Discussion

Patients undergoing bariatric surgery overall responded as expected to the surgical procedure with a massive weight loss and an overall amelioration of risk factors one year after surgery. In addition, bariatric surgery affected the number and composition of extracellular vesicles in general. The reduction of extracellular vesicles was due to a reduction in Annexin V^–^ extracellular vesicles. Nonetheless, extracellular vesicles derived from the liver were reduced overall regardless of Annexin V staining. This finding was supported by analyzing liver specific mRNA within the pool of extracellular vesicle derived RNA. Similarly, to the reduction observed using specific antibodies in flow cytometry, liver specific mRNA content was reduced in circulating extracellular vesicles after bariatric surgery.

Extracellular vesicles that are produced by an active budding process from the cellular membrane of the parental cell allow a direct tracing of cellular origin by using proteins specific for the parent cell [19]. The antigen for HepPar1 used to identify hepatic vesicles was identified to be CPS1, a rate limiting enzyme in the urea cycle, which is abundantly expressed in the liver [20]. Even though CPS1 is not a receptor, extracellular vesicles contained CPS1 probably by incorporation of the cytoplasm during budding as suggested previously [21]. ASGPR is an abundant hepatocyte-specific carbohydrate that binds glycoproteins lacking terminal sialic acid residues [11]. In our study hepatic extracellular vesicle populations showed a significant decrease after bariatric surgery suggesting a regulation of vesicle production rather than a conserved steady state process. Previously, Povero et al. already reported increased ASGPR^+^ circulating microvesicles in a rat model of liver injury and fatty liver disease [9]. Within our cohort number of vesicles or changes in vesicles’ characteristics did not correlate with clinical parameters of liver function or inflammation besides Annexin V^+^ ASGPR^+^ extracellular vesicles which correlated with ALT. However, PS^–^ vesicles correlated with levels of PAI-1. It should be noted that PAI-1 was previously shown to correlate with liver dysfunction [22]. We therefore speculate that most liver specific extracellular vesicles represent a so far unmeasured laboratory parameter of liver function not represented in standard laboratory parameters. 

**Figure 4:**
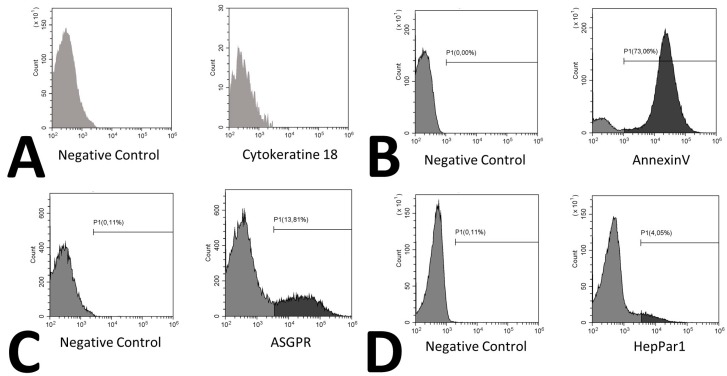
Histograms of extracellular vesicle staining. Histograms for cytokeratine 18 labeled with FITC (**A**), Annexin V labeled with PE-Cy7 (**B**), ASGPR labeled with APC (**C**), and HepPar1 labeled with PE (**D**) are shown. Histograms from Annexin V, ASGPR and HepPar1 are derived from the same donor.

Recently, hepatic microvesicles were shown to predict mortality in patients with cirrhosis using cytokeratin 18 as a marker for hepatic vesicles [23]. These cytokeratin 18 positive vesicles are however only detectable in patients with cirrhosis and were measured by an ELISA based assay [24]. In addition, cytokeratin 18 is also associated with apoptosis of liver cells in cirrhosis [25]. Within our cohort, even though patients were morbidly obese, liver dysfunction was only moderate as demonstrated by AST, ALT and GGT levels. Similar to the previously undetectable nature of cytokeratin 18 extracellular vesicles in healthy control patients by ELISA, we were not able to detect cytokeratin 18 positive vesicles when using a directly labeled antibody in flow cytometry (Figure 4A). We therefore suggest that ASGPR and HepPar1 antigens might be suitable markers for hepatic vesicles in patients without cirrhotic liver dysfunction. 

In addition, as vesicles have been shown to be loaded with mRNA from their host cells [26] and abundant mRNAs in vesicles are also highly expressed in parental cells, we analyzed four mRNAs encoded by genes that are hepatocyte specific [27,28]. Overall, detection of multiple mRNAs after bariatric surgery was reduced. Whereas three liver specific mRNAs were detectable in circulating vesicles in 48% of the patients before surgery this dropped to 18% after surgery. The majority of patients had only one to two miRNAs present in the extracellular vesicle fraction suggesting a reduction in mRNA levels within the total pool of RNA isolated from extracellular vesicles. As total RNA content and the expression of two housekeeping genes in extracellular vesicles was unaltered after bariatric surgery, we suggest that the observed reduction in liver specific mRNA is due to a reduction of liver specific circulating extracellular vesicles as demonstrated by flow cytometry.

In summary we conclude that circulating hepatic extracellular vesicles are detectable in samples from patients undergoing gastric bypass surgery. Even though we can speculate on applications in clinical practice, the aim of our research was to demonstrate the proof of principal that detecting extracellular vesicles in the circulation is possible in patients before and after massive weight loss. We further demonstrate that this was achieved by using two different markers, ASGPR and HepPar1 instead of previously demonstrated cytokeratin 18, which is only detectable at late stages of cirrhosis. Similarly, to the observed amelioration of liver markers AST, ALT and GTT, hepatic vesicles are reduced following weight loss. We therefore suggest that ASGPR or HepPar positive vesicles might be potential candidates as liver specific vesicle markers.

### Limitations

There are several limitations in this exploratory study. Foremost, only patients undergoing RYGBP surgery were included in the study. Furthermore, we did not include a healthy control group or a morbidly obese group to determine basal fluctuations in hepatic medium EVs. This surgery showed a beneficiary effect in all 27 analyzed patients. Furthermore, weight loss remained constant after one and also after two years after RYGBP surgery. Therefore, a stratification of patients for efficiency cannot be performed. We would also like to speculate that none of our patients was metabolically healthy before surgery as all patients showed a beneficial effect of RYGBP surgery. Lack of correlation could be due to the small sample size of 27 in addition with a gender disbalance. Furthermore, we found differences in PS^+^ extracellular vesicle behavior when comparing flow cytometry and ELISA results. Whereas flow cytometry suggested a trend for a decline of PS^+^ extracellular vesicles, ELISA results did not show the same trend. This could be due to the different detection methods used. In flow cytometry we used Annexin V to detect vesicles whereas in the ELISA system an indirect assay via capturing of the vesicles using Annexin V is employed and subsequently the activation of the FXa–FVa system via these captured vesicles is measured by the capability to activate prothrombin into thrombin. This indirect system could potentiate the actual number as it is more dependent on the total amount of PS on the vesicle surface which might lead to the documented differences. 

## 4. Materials and Methods

### 4.1. Study Population

Twenty-seven severely obese patients with a BMI >40 undergoing RYGBP were enrolled in the study. Citrated blood was drawn before surgery and one year after. Platelet poor plasma was obtained by centrifugation at 1500× *g* for 20 min [29]. Samples were stored at −80 °C in multiple aliquots. All human material was obtained and processed according to the recommendations of the hospital’s ethics committee and security board, including informed consent. The endpoint of this observational study was one year after surgery. Data was available for all 27 patients. As previously published, patients had a massive weight loss one year after bariatric surgery, but no complications were reported for our included patients [16]. Therefore, the endpoint of our study was set for plasma parameters one year after RYGBP. This study was approved by the Ethical Board Burgenland KRAGES, on 11 February 2011. The identification code of the project is 39/2011. 

### 4.2. Laboratory Parameter Determination

AST, ALT, and GGT were determined using routine assays on a cobas^®^ 501 instrument (Roche Diagnostics, Basel, Switzerland). Concentrations of highly sensitive CRP (hs-CRP) were measured using particle enhanced immunoturbidimetric assay on cobas^®^ 8000 modular analyzer (Cardiac C-Reactive Protein (Latex) High Sensitive, Roche Diagnostics, Basel, Switzerland). PAI-1 levels were determined using a commercially available ELISA (Technoclone, Vienna, Austria).

### 4.3. Isolation of Extracellular Vesicles

For isolating particles prior to staining 100 µL of plasma samples were centrifuged at 18,000× *g* for 30 min to pellet extracellular vesicles. Vesicles were stained immediately as washing was shown to not change extracellular vesicle number and content [29].

### 4.4. Determination of Extracellular Vesicles

**Figure 5:**
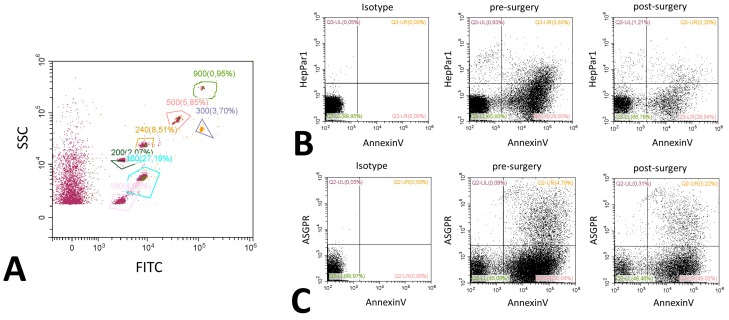
Representative flow cytometry dot plots. Dot plots for bead-based size estimation showing a bead mix labeled with FITC and an unlabeled fraction (**A**). The various gates are labeled with the size of the measured beads, percentages represent the relative number of beads measured compared to the whole sample. Dot plots are further shown for Annexin V and HepPar1 (**B**) and ASGPR and Annexin V (**C**). All examples are given before and after surgery. A representative isotype control picture is included in the panel. Gating was performed according to the values obtained in the histograms, percentages display the relative amount of events in comparison to the total event number.

Extracellular vesicles were analyzed using flow cytometry on a Cytoflex (Beckman Coulter, Brea, CA, USA) [30]. Isolation and staining of vesicles were performed as described by Chiva-Blanch et al. previously [31]. Values are given as events/100 µL of plasma. Vesicles were defined as being between 200 nm to 900 nm of size according to size specific fluorescence beads (Megamix Plus, Biocytex, Marseille, France) and initial gating was solely based on size. Therefore, and in accordance with the guidelines, we termed this fraction medium EVs [7]. Hepatic extracellular vesicles were identified using a mouse monoclonal anti-human-hepatocyte marker (HepPar1, Clone OCH1E5, Dako, Carpinteria, CA, USA) or a mouse monoclonal anti-human- ASPGR antibody (BD, Franklin Lakes, NJ, USA). Both antibodies were directly labeled using a specific kit (Abcam, Cambridge, UK) with APC labeling for ASGPR and PE labeling for HepPar1. PS was stained using an AnnexinV conjugate (Thermo Fisher, Waltham, MA, USA, PE-Cy7), cytokeratine 18 was stained using an anti-human-cytokeratine 18 FITC prelabeled monoclonal mouse flow cytometry antibody (Thermo Fisher, Waltham, MA, USA). After staining, samples were washed to remove excess antibodies. In order to determine the control and background fluorescence we followed the suggestions from Hulspas et al. [32] including unstained but fully processed samples labeled as negative controls, and Boolean gating strategy during the establishment of the staining protocol. Example histograms of stainings are shown in Figure 4, an example for bead sizing and representative dot plots for ASGPR and HepPar1 are shown in Figure 5.

### 4.5. ELISA Determination of Annexin V^+^ Vesicles

Annexin V can be used to stain PS positive cells and extracellular vesicles. We used a commercially available ELISA kit (Hyphen Biomed, Neuville-sur-Oise, France) to determine the concentration of Annexin V^+^ extracellular vesicles in the circulation according to the manufacturer’s instructions. In short, Annexin V^+^ extracellular vesicles were captured using an Annexin specific antibody. Extracellular vesicle amount was determined indirectly via measuring the activity of FXa–FVa to activate prothrombin into thrombin.

### 4.6. RNA Isolation and qPCR

RNA was isolated from the pellet formed by extracellular vesicles after centrifugation of 250 µL plasma at 18,000 rpm for 20 min using an automated system (Maxwell, Promega, Madison, WI, USA) and the respective kit (Maxwell^®^ 16 LEV simplyRNA, Promega, Madison, WI, USA) including a spike in control to guarantee equal extraction (TATAAbiocenter, Gothenburg, Sweden). Total RNA was quantified fluorimetrically (Quantus, Promega, Madison, WI, USA). cDNA was generated from RNA using a Promega GoScript reverse transcription system (Promega, Madison, WI, USA). qPCR was performed on a LightCycler 480 system (Roche, Basel, Switzerland) using GoTaq Probe qPCR master mix (Promega, Madison, WI, USA). Primers were designed using the Universal Probe Library (UPL) primer design program (Roche, Basel, Switzerland). Primer sequences and used probes were as follows, acyl-coenzyme-A-oxidase-1 (ACOX) forward 5′—acagtcctactgtgacctccatt—3′ reverse 5′—ttgcatgatttgaagtctttcc—3′, UPL probe 49; acyl-coenzyme-A-dehydrogenase (ACADM) 5′—aggagccattgatgtgtgc—3′, reverse 5—ctgctttggtctttataccagcta—3′, UPL probe 1; hydroxyacyl-coenzyme-A-dehydrogenase (HADH) forward 5′—ctcggccaagaagataatcg—3′, reverse 5′—tctaccaacactactgtgtgacca-3′, UPL probe 61; 3-hydroxybutyrate-dehydrogenase (BDH) forward 5′- ttgctggctgcttgatga—3′, reverse 5′—tcaatcggtcactgtttaggc—3′, UPL probe 17; glyceraldehyde-3-phosphate-dehydrogenase (GAPDH) forward 5′—agccacatcgctcagacac—3′, reverse 5′—gcccaatacgaccaaatcc—3′, UPL probe 60; 18s forward 5′—gcaattattccccatgaacg—3′, reverse 5′— gggacttaatcaacgcaagc—3′, UPL probe 48. Spike in control was measured using available primers from TATAAbiocenter. Of note, we did not detect positive signals in 8 EV depleted plasma samples above the threshold suggesting that the majority of mRNA originated from EVs.

### 4.7. Statistical Analysis

Sample parameters did not show a normal distribution as determined by a Kolmogorov–Smirnov-test, therefore we used a Wilcoxon rank test to determine significance for laboratory data, cytokine analysis and all flow cytometry data. A chi square test was used to demonstrate a significant change in mRNA distribution analysis. Calculations were performed using SPSS21, *p* ≤ 0.05 was considered significant. Percent reduction was calculated using the median values, patients’ characteristics are given as a mean to allow easy comparison with the literature.

## Figures and Tables

**Table 1:** Patient characteristics.

**Total Patients**	27	
**Male**	6 (22%)	
**Female**	21 (78%)	
**Age**	43 ± 13	
**Diabetes**	8 (30%)	
**Smoking**	8 (30%)	
	**before surgery**	**one year after surgery**
**AST U/L**	28.44 ± 13.9	22.28 ± 12.3 (*p* = 0.054)
**ALT U/L**	42.96 ± 28.1	21.47 ± 11.6 (*p* < 0.001)
**GGT U/L**	62.96 ± 72.3	21.18 ± 45.4 (*p* < 0.001)
**HDL (mg/dL)**	48.9 ± 14.4	55.8 ± 5.3 (*p* = 0.013)
**LDL (mg/dL)**	103.2 ± 24	76.9 ± 20.5 (*p* < 0.001)
**CRP (mg/dL)**	0.924 ± 0.8	0.414 ± 1.1 (*p* < 0.001)
**PAI-1 (ng/mL)**	99 ± 14.6	86.7 ± 27 (*p* = 0.048)
**Medication**		
**Statins**	7 (26%)	2 (8%)
**Antidiabetics**	6 (22%)	2 (8%)
**Insulin**	3 (11%)	2 (8%)
**ACE inhibitors**	14 (52%)	8 (30%)
**Beta blocker**	6 (22%)	2 (8%)

Overall patient characteristics including medication before and after surgery are shown. Statistical significance was calculated using Wilcoxon test. *p* ≤ 0.05 was considered significant.

## Supplementary Materials

Supplementary materials can be found at https://www.mdpi.com/1422-0067/20/9/2153/s1.
