# Supplementary material for: Changes of Circulating Extracellular Vesicles from the Liver after Roux-en-Y Bariatric Surgery
Source: Int J Mol Sci. 2019 Apr 30;20(9):2153. doi: 10.3390/ijms20092153 (PMC6539504; doi:10.3390/ijms20092153)
Supplement: Supplementary file 1 [file ijms-20-02153-s001.pdf]

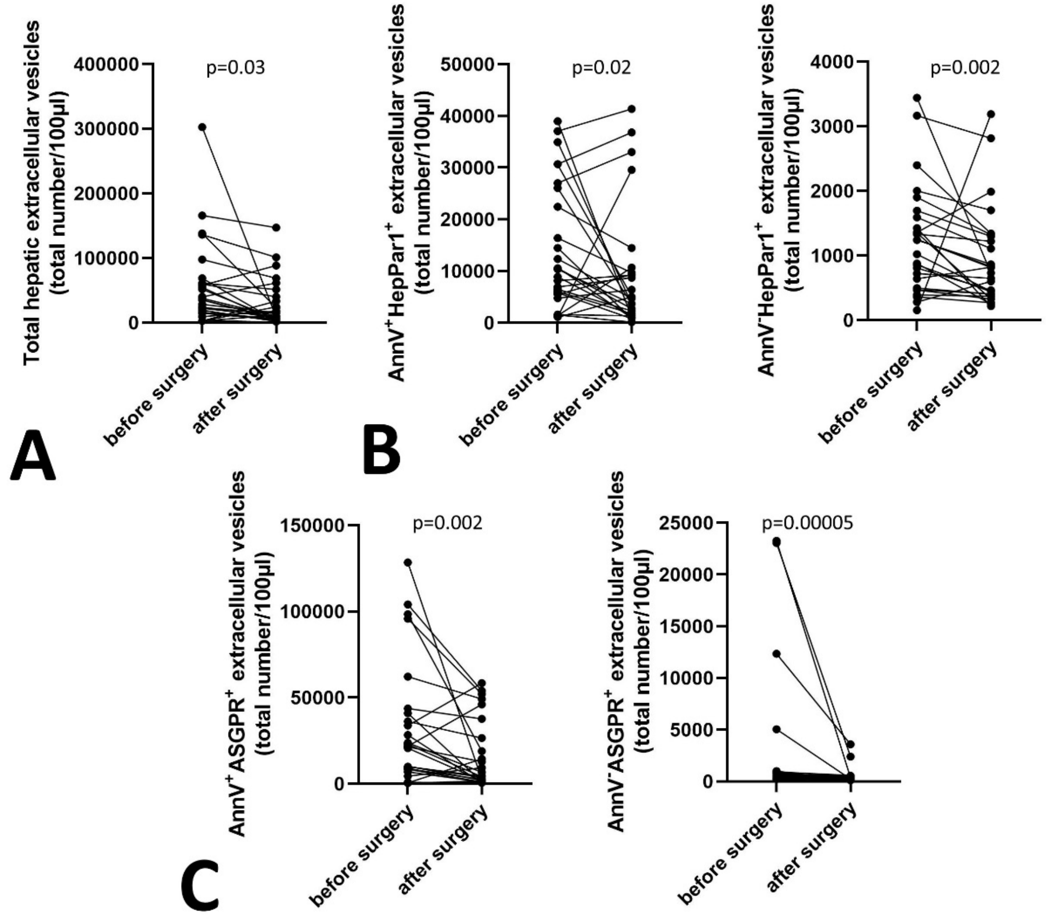

Supplementary Figure 1:

Individual data points for all patients are given for total hepatic EVs (A), HepPar1 EVs (B), and ASGPR EVs (C).
